# Supplementary material for: A numerical model of the MICP multi-process considering the scale size
Source: PLoS One. 2024 Jan 29;19(1):e0297195. doi: 10.1371/journal.pone.0297195 (PMC10824416; doi:10.1371/journal.pone.0297195)
Supplement: Attachment — Submitted filename: PONE-D-23-20748_reviewer (5).pdf [file pone.0297195.s004.pdf]

PLOS ONE

# A numerical model of the MICP multi-process considering the scale size

--Manuscript Draft--

| | |
|---|---|
| Manuscript Number: | PONE-D-23-20748 |
| Article Type: | Research Article |
| Full Title: | A numerical model of the MICP multi-process considering the scale size |
| Short Title: | A numerical model of the MICP multi-process considering the scale size |
| Corresponding Author: | Yujie Li<br>Zhejiang University<br>Zhoushan, CHINA |
| Keywords: | MICP; Multi-process; Convective diffusion reaction; Numerical model; Scale size; Solute transport |
| Abstract: | As an environmentally friendly and controllable technology, Microbially induced carbonate precipitation (MICP) has broad applications in geotechnical and environmental fields. However, the longitudinal dispersivity in MICP multi-process varying with the scale size. Ignoring the effect of the scale size of the research object on the dispersivity leads to the inaccuracy between the numerical model and the experiment data. Thus, this paper established the relationship between the scale size and the dispersivity initially, and optimized the theoretical system of MICP multi-process reaction. Results show that: 1) The relationship between the dispersivity and the scale size presented as a substantially linear relationship in the double logarithmic coordinate system. Considering the size effect is closer to the experimentally measured data. 2) After considering the scale size, the suspended and attached bacteria are higher than that without considering the size effect, which leading to a higher calcium carbonate content. 3) The maximum carbonate content increases with the increase of the initial porosity, and the average carbonate showed a significant increasing trend with the increase of the bacterial injecting rate. Both the smaller initial porosity and higher bacterial injecting rate are more conducive to the uniform distribution of the calcium carbonate. In the simulation of the microbial mineralization kinetic model, it is recommended to consider the influence of the scale size on the MICP multi-process. |
| Order of Authors: | Xianxian Zhu |
| | Jianhua Wang |
| | Haili Wang |
| | Yujie Li |
| Additional Information: | |
| Question | Response |
| **Financial Disclosure**<br><br>Enter a financial disclosure statement that describes the sources of funding for the work included in this submission. Review the submission guidelines for detailed requirements. View published research articles from *PLOS ONE* for specific examples.<br><br>This statement is required for submission and **will appear in the published article** if the submission is accepted. Please make sure it is accurate. | The authors would like to acknowledge the supports by the Zhejiang Water Conservancy Key Science and Technology Project (RB2120), Hainan Special PhD Scientific Research Foundation of Sanya Yazhou Bay Science and Technology City (HSPHDSRF-2022-04-002). |

Powered by Editorial Manager® and ProduXion Manager® from Aries Systems Corporation

| | |
|---|---|
| **Unfunded studies**<br>Enter: *The author(s) received no specific funding for this work.*<br><br>**Funded studies**<br>Enter a statement with the following details:<br>• Initials of the authors who received each award<br>• Grant numbers awarded to each author<br>• The full name of each funder<br>• URL of each funder website<br>• Did the sponsors or funders play any role in the study design, data collection and analysis, decision to publish, or preparation of the manuscript?<br>• **NO** - Include this sentence at the end of your statement: *The funders had no role in study design, data collection and analysis, decision to publish, or preparation of the manuscript.*<br>• **YES** - Specify the role(s) played.<br><br>* typeset | |
| **Competing Interests**<br><br>Use the instructions below to enter a competing interest statement for this submission. On behalf of all authors, disclose any competing interests that could be perceived to bias this work—acknowledging all financial support and any other relevant financial or non-financial competing interests.<br><br>This statement is required for submission and **will appear in the published article** if the submission is accepted. Please make sure it is accurate and that any funding sources listed in your Funding Information later in the submission form are also declared in your Financial Disclosure statement.<br><br>View published research articles from *PLOS ONE* for specific examples. | The authors have declared that no competing interests exist. |

| | |
|---|---|
| **NO authors have competing interests**<br><br>Enter: *The authors have declared that no competing interests exist.*<br><br>**Authors with competing interests**<br><br>Enter competing interest details beginning with this statement:<br><br>*I have read the journal's policy and the authors of this manuscript have the following competing interests: [insert competing interests here]*<br><br>* typeset | |
| **Ethics Statement**<br><br>Enter an ethics statement for this submission. This statement is required if the study involved:<br><br>• Human participants<br>• Human specimens or tissue<br>• Vertebrate animals or cephalopods<br>• Vertebrate embryos or tissues<br>• Field research<br><br>Write "N/A" if the submission does not require an ethics statement.<br><br>General guidance is provided below. Consult the submission guidelines for detailed instructions. **Make sure that all information entered here is included in the Methods section of the manuscript.** | N/A |

Powered by Editorial Manager® and ProduXion Manager® from Aries Systems Corporation

| | |
|---|---|
| **Format for specific study types**<br><br>**Human Subject Research (involving human participants and/or tissue)**<br>• Give the name of the institutional review board or ethics committee that approved the study<br>• Include the approval number and/or a statement indicating approval of this research<br>• Indicate the form of consent obtained (written/oral) or the reason that consent was not obtained (e.g. the data were analyzed anonymously)<br><br>**Animal Research (involving vertebrate animals, embryos or tissues)**<br>• Provide the name of the Institutional Animal Care and Use Committee (IACUC) or other relevant ethics board that reviewed the study protocol, and indicate whether they approved this research or granted a formal waiver of ethical approval<br>• Include an approval number if one was obtained<br>• If the study involved *non-human primates*, add *additional details* about animal welfare and steps taken to ameliorate suffering<br>• If anesthesia, euthanasia, or any kind of animal sacrifice is part of the study, include briefly which substances and/or methods were applied<br><br>**Field Research**<br><br>Include the following details if this study involves the collection of plant, animal, or other materials from a natural setting:<br>• Field permit number<br>• Name of the institution or relevant body that granted permission | |
| **Data Availability**<br><br>Authors are required to make all data underlying the findings described fully available, without restriction, and from the time of publication. PLOS allows rare exceptions to address legal and ethical concerns. See the PLOS Data Policy and FAQ for detailed information. | Yes - all data are fully available without restriction |

| | |
|---|---|
| A Data Availability Statement describing where the data can be found is required at submission. Your answers to this question constitute the Data Availability Statement and **will be published in the article**, if accepted.<br><br>**Important:** Stating 'data available on request from the author' is not sufficient. If your data are only available upon request, select 'No' for the first question and explain your exceptional situation in the text box.<br><br>Do the authors confirm that all data underlying the findings described in their manuscript are fully available without restriction? | |
| **Describe where the data may be found in full sentences. If you are copying our sample text, replace any instances of XXX with the appropriate details.**<br><br>• If the data are **held or will be held in a public repository**, include URLs, accession numbers or DOIs. If this information will only be available after acceptance, indicate this by ticking the box below. For example: *All XXX files are available from the XXX database (accession number(s) XXX, XXX.*).<br>• If the data are all contained **within the manuscript and/or Supporting Information files**, enter the following: *All relevant data are within the manuscript and its Supporting Information files.*<br>• If neither of these applies but you are able to provide **details of access elsewhere**, with or without limitations, please do so. For example:<br><br>*Data cannot be shared publicly because of [XXX]. Data are available from the XXX Institutional Data Access / Ethics Committee (contact via XXX) for researchers who meet the criteria for access to confidential data.*<br><br>*The data underlying the results presented in the study are available from (include the name of the third party* | All relevant data are within the manuscript and its Supporting Information files. |

| | |
|---|---|
| *and contact information or URL).*<br>• This text is appropriate if the data are owned by a third party and authors do not have permission to share the data.<br><br>* typeset | |
| Additional data availability information: | |

Manuscript

Click here to access/download;Manuscript;manuscriptV3.docx

# A numerical model of the MICP multi-process considering the scale size

Zhu Xianxian[1], Wang Jianhua[1], Wang Haili[2], Li Yujie[3,*]

1 Zhejiang Qiantang River Basin Center, Hangzhou 310016, China.

2 Survey and Design Institute of Qiantang River Administration Bureau of Zhejiang Province, Hangzhou310016, China.

3 Ocean College, Zhejiang University, Zhoushan 316021, China.

* Corresponding author, Email: liyujies@163.com. Tel: +86 15671559761.

**Abstract:** As an environmentally friendly and controllable technology, Microbially induced carbonate precipitation (MICP) has broad applications in geotechnical and environmental fields. However, the longitudinal dispersivity in MICP multi-process vary[illegible]g with the scale size. Ignoring the effect of the scale size of the research object on the dispersivity leads to the inaccuracy between the numerical model and the experiment data. Thus, this paper [illegible]blished the relationship between the scale size and the dispersivity initially, and optimized the theoretical system of MICP multi-process reaction. Results show[illegible]at: 1) The relationship between the dispersivity and the scale size presented as a substantially linear relationship in the double logarithmic coordinate system. Considering the size effect is closer to the experimentally measured data. 2) After considering the scale size, the suspended and attached bacteria are higher than that without considering the size effect, which leading to a higher calcium carbonate content. 3) The maximum carbonate content increases with the increase of the initial porosity, and the average carbonate showed a significant increasing trend with the increase of the bacterial injecting rate. Both the smaller initial porosity and higher bacterial injecting rate are more conducive to the uniform distribution of the calcium carbonate. In the simulation of the mic[illegible]ial mineralization kinetic model, it is recommended to consider the influence of the scale size on the MICP multi-process.

**Keywords:**

MICP; Multi-process; Convective diffusion reaction; Numerical model; Scale size; Solute transport

## 1. Introduction

As shown in Fig. 1, microbially induced carbonate precipitation (MICP) has attracted extensive attention in coastal zone reinforcement (Kou et al., 2020; Salifu et al., 2016; Yu et al., 2022), erosion and scour protection (Xiao et al., 2022; Li et al., 2022; Tao et al., 2018; Jiang et al. 2017), ground

improvement (Xiao et al., 2018; Xiao et al., 2019) and pile foundation reinforcement (Xiao et al., 2021; Li et al., 2020). Among them, urea is decomposed by *Sporosarcina pasteurii* into ammonium and calcium, which combines with carbonate ion to produce calcium carbonate, leading to the particle coating, particle cementing, pore filling in sand (Burbank et al., 2011; Whiffin et al., 2007; Mujah et al., 2019; Alexandra et al., 2020; Song et al., 2022; Fang et al., 2021; Zeng et al., 2021; Mohamed et al., 2017; Lyu et al., 2022; Kashizadeh et al., 2021; Huang et al., 2022; Bu et al., 2022). The chemical reaction is shown as below.

$$CO(NH_2)_2 + 2H_2O \xrightarrow{bacteria/urease} 2NH_4^+ + CO_3^{2-} \tag{1}$$

$$CO_3^{2-} + Ca^{2+} \rightarrow CaCO_3 \downarrow \tag{2}$$

The MICP reaction in sand is very complex, involving multiple processes under the coupling of physical-chemical-biological fields. The injecting rate directly affects the progress of the MICP reaction. As shown in Fig. 1, the flow velocity distribution of pore fluid in sand mainly includes three forms: 1) the uneven flow velocity distribution in single pore, the maximum flow velocity appears in the middle of the pore due to the influence of particle friction; 2) Split flow in multiple pores, the difference in pore size and shape leads to the difference in flow velocity; 3) Lateral dispersion caused by particle blocking, which is the main factor causing the lateral dispersion of the solute. Importantly, when the MICP reaction occurs in the sand, the deposited calcium carbonate encapsulates the particles, plugs pores, and connects the particles, which all alter the flow velocity distribution of the pore. The pore diameter decreases when calcium carbonate is deposited on the particle surface, resulting in an increase in pore velocity. In addition, the solute (urea, calcium, ammonium and bacteria) is transported over long distances along the flow direction, while the diffusion still appears perpendicular to the direction of the flow. As a result, the concentration of the solute exhibits a non-uniform distribution characteristic similar to an ellipse over time.

Up to now, most of the research about MICP has focused on the lab experiments. The spatiotemporal evolutions of various chemical substances in MICP multi-process cannot be obtained in real time. The field experiments cost a lot of manpower and resources, and are highly dependent on the field conditions. Therefore, reliable model is of great significance for understanding biochemical processes in MICP reaction (Fauriel et al., 2012; Wang et al., 2020a; Nassar et al., 2018).

The convection-diffusion-reaction are always used to describe the evolutions of the suspended bacteria, urea, ammonium, and calcium in MICP numerical model. Hydrodynamic dispersion is the main way of the substance migration in sand, including the molecular diffusion caused by concentration gradients and the mechanical dispersivity caused by pore flows. Dispersion is significantly directional, dependent on pore characteristics and scale size. According to Gelhar (1992), the longitudinal dispersivity, which is the most difficult parameter to determine in solute transport, ranged from $10^{-2}$ to $10^{4}$ m for size ranging from $10^{-1}$ to $10^{5}$ m. This value cannot be directly measured. The results of the Toride's (2003) study show that the dispersivity does not change with the velocity of the pore flow. Godoy (2018) found that the longitudinal dispersivity have significant size effects with assuming a liner relationship between the longitudinal dispersivity and the flow velocity. However, the hydrodynamic dispersivity coefficient was mostly taken as the molecular dispersivity coefficient in the simulation of MICP multi-process, the mechanical dispersivity and the effect of the pore tortuosity on the molecular dispersivity were always ignored (Nishimura et al., 2021; Von Wolff et al., 2021; Zeng et al., 2021). Some scholars have divided hydrodynamic dispersivity into mechanical dispersivity and molecular dispersivity in a more detailed way, but the values of dispersivity are quite different (Wang et al., 2020a; Hommel et al., 2015; D.Landa-M. et al., 2021; Minto et al., 2019). As shown in Fig.2, the scale of the research objects for the MICP technology ranges from micro to macro, but the influence of the scale size on the dispersivity in MICP multi-process has not yet been reported. There is no unified way for the value of the hydrodynamic dispersivity in the numerical model of the MICP multi-process, and improper handling leads to the inaccuracy between the model and the experimental data.

Thus, this study established the relationship between the scale size and the longitudinal dispersivity initially, and optimized the MICP multi-process numerical model. The influences of the scale size on the dispersivity, bacteria and calcium carbonate distribution in MICP multi-process were analysed. In addition, the parametric sensitivity analysis was also performed for initial porosity and bacterial injecting rate.

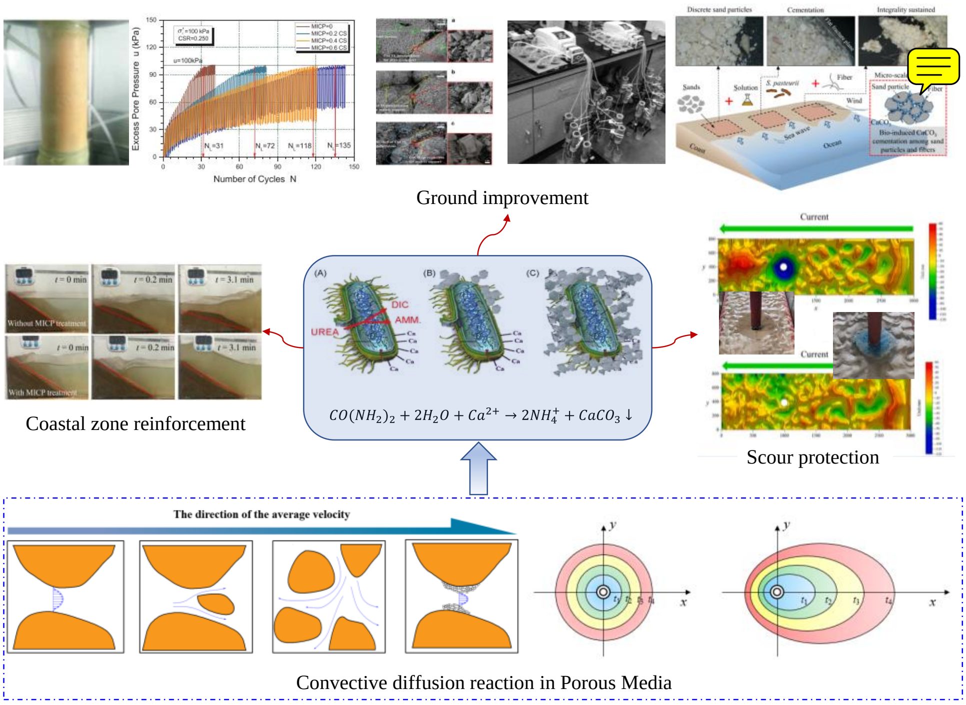


**Fig. 1** The MICP application scenarios and the schematic diagram of convective diffusion reaction.

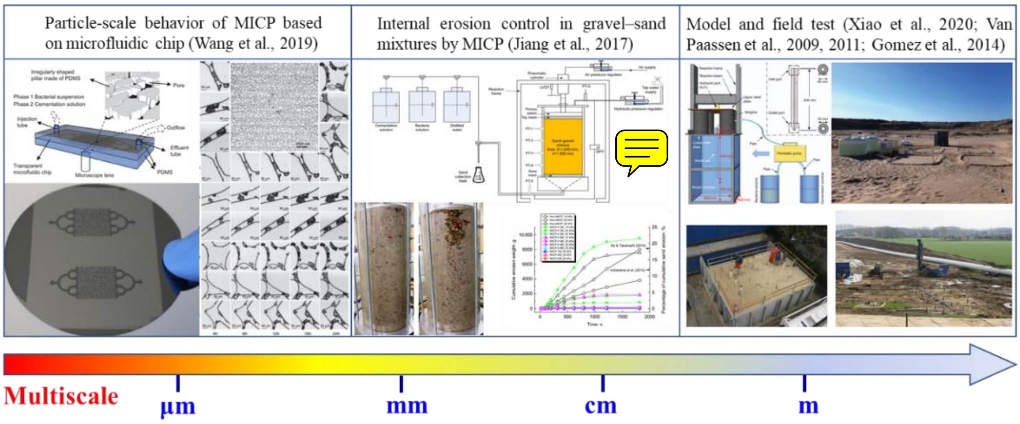


**Fig. 2** Multiscale applications of MICP technology.

## 2. Mathematical model of the MICP multi-process

### *2.1 Multi-stages of MICP grouting*

Biomineralization in sand involves multi-process of physical-chemical-biological reaction. It can be divided into: 1) Bacteria injection stage (Phase B), the convection and diffusion of the suspended bacteria appear in sand, and the attached bacteria accumulate on the particle surface; 2) bacteria retention stage (Phase R), bacteria show molecular diffusion and adsorption; 3) Cementing solution injection stage (Phase C), this stage includes the convective diffusion reaction of all solutes, the formation of the calcium carbonate, the reduction in porosity and the permeability of the sand. Due to the consumption of the substances and the encapsulation of the calcium carbonate, the decay of the

bacteria is considered throughout the process.

### *2.2 The bacterial behaviour*

Bacteria shows the growth, decay and attachment in sand. Due to the interception and scree of particles, some bacteria adhere to the particle surface ($C_{\text{bacs}}$), while some bacteria are free in the pore and migrate with the fluid ($C_{\text{bacl}}$). The number and activity of the bacteria gradually weakens because of the lack of the nutrient supply and the encapsulation of the calcium carbonate. In generally, the first-order kinetic equation is used to describe the behaviour of bacteria in sand (Wang et al., 2020a). The equations are shown below.

$$\frac{\partial C_{bact}}{\partial \mathrm{t}} = -k_{\mathrm{d}} C_{bact} \tag{3}$$

$$\frac{\partial C_{bacs}}{\partial \mathrm{t}} = k_{\mathrm{att}} C_{bacl} - k_{\mathrm{d}} C_{bacs} \tag{4}$$

where $k_{\mathrm{d}}$ is a constant decay rate, and $k_{\mathrm{att}}$ is the constant attachment rate. The detail is shown in Li et al., (2023), the author established the kinetic theory of biomineralization in seabed under the action of waves, and this paper focuses on analysing the influence of scale size on the biomineralization reaction in sand.

### *2.3 The fluid equations and solute transport diffusion equations*

Considering that the fluid is incompressible, the continuity equation is used to describe the relationship between fluid pressure and flow velocity (Eq. 4). It is worth noting that the fluid density changes during the mineralization reaction, as shown in Eq. 5.

$$\frac{d(\varphi\rho_{\mathrm{l}})}{dt} = -\nabla\cdot(\rho_{\mathrm{l}} q) - m_{\mathrm{urea}}\varphi k_{\mathrm{rea}} - m_{\mathrm{Ca^{2+}}}\varphi k_{\mathrm{rea}} + 2m_{\mathrm{NH_4^+}}\varphi k_{\mathrm{rea}} \tag{4}$$

where $\varphi$, $m_{\mathrm{urea}}$, $m_{\mathrm{Ca^{2+}}}$, $m_{\mathrm{NH_4^+}}$ represent the porosity, molar mass of urea, calcium and ammonium, respectively. $k_{\mathrm{rea}}$ is the urea hydrolysis constant, determined by Eq. 14.

$$\rho_l = \rho_w + 0.0154994\, kg/mol \cdot C_{urea} + 0.0867338\, kg/mol \cdot C_{Ca^{2+}} + 0.0158991\, kg/mol \cdot C_{NH_4^+} \tag{5}$$

where $\rho_w$, $C_{urea}$, $C_{Ca^{2+}}$, $C_{NH_4^+}$ are the water density, substance concentration of urea, calcium and ammonium, respectively.

Assuming that the system is laminar flow conditions, the relationship between the average velocity $q$ and liquid pressure $p_l$ can be described by Darcy's law (Eq. 5).

$$q = -\frac{K}{\mu_l} \cdot (\nabla p_l + \rho_l \boldsymbol{g}) \quad (6)$$

where $K$ is the matrix permeability, $\boldsymbol{g}$ is the gravity acceleration, the $\mu_l$ and $\rho_l$ represent the liquid viscosity and the density.

The general form of the convective-diffusion-reaction is as follows:

$$\varphi \frac{\partial C_{urea}}{\partial t} = \nabla \cdot (\varphi \boldsymbol{D}^* \cdot \nabla C_{urea}) - q \cdot \nabla C_{urea} - \varphi k_{rea} \quad (7)$$

$$\varphi \frac{\partial C_{\mathrm{NH_4^+}}}{\partial t} = \nabla \cdot \left(\varphi \boldsymbol{D}^* \cdot \nabla C_{\mathrm{NH_4^+}}\right) - q \cdot C_{\mathrm{NH_4^+}} + 2\varphi k_{\mathrm{rea}} \quad (8)$$

$$\varphi \frac{\partial C_{\mathrm{Ca^{2+}}}}{\partial t} = \nabla \cdot (\varphi \boldsymbol{D}^* \cdot \nabla C_{\mathrm{Ca^{2+}}}) - q \cdot C_{\mathrm{Ca^{2+}}} - \varphi k_{\mathrm{rea}} \quad (9)$$

$$\varphi \frac{\partial C_{\mathrm{bacl}}}{\partial t} = \nabla \cdot (\varphi \boldsymbol{D}^* \cdot \nabla C_{\mathrm{bacl}}) - q \cdot C_{\mathrm{bacl}} - \varphi k_{\mathrm{d}} C_{\mathrm{bacl}} - \varphi k_{\mathrm{att}} C_{\mathrm{bacl}} \quad (10)$$

where $C_i$ represents the concentration of the urea, calcium, ammonium and suspended bacteria, $\boldsymbol{D}^*$ represent the hydrodynamic dispersivity coefficient in sand. Solute diffusion in saturated sand mainly includes mechanical diffusion and molecular diffusion (Bear 1972), so there are:

$$\boldsymbol{D}^* = (\alpha_L - \alpha_T)\frac{v \otimes v}{|v|} + \alpha_T |v| \boldsymbol{I} + \frac{\varphi}{\tau}\boldsymbol{D} \quad (11)$$

where, $\boldsymbol{D}^*$ and $\boldsymbol{D}$ represent the hydrodynamic dispersivity coefficient in sand and water, $\alpha_L$ and $\alpha_T$ are the vertical and horizontal dispersivity, $v$ is the pore water velocity ($q/\varphi$) and $\tau$ represents the tortuosity, the Millington-Quirk model is used to describe the tortuosity in this paper.

$$\tau = \varphi^{-1/3} \quad (12)$$

When the research object is one-dimensional condition, Eq. 11 degenerates into:

$$\boldsymbol{D}^* = \alpha_L \cdot v + \frac{\varphi}{\tau}\boldsymbol{D} \quad (13)$$

The value of the hydrodynamic dispersivity coefficient $\boldsymbol{D}$ is taken as 2e-9 $m^2/s$ in this paper. According to Eq. 1, consuming 1 mol urea will produce 2 mol calcium in urea hydrolysis. The research of Whiffin (2004) show that the ammonium has no obvious inhibition on urease activity. Therefore, the Monod equation (Fauriel et al., 2012; Van Wijingaarden et al., 2011) is used to describe the kinetics of urea hydrolysis.

$$k_{rea} = u_{sp}(C_{bacs} + C_{bacl}) \cdot \frac{C_{urea}}{C_{urea} + k_m} \cdot \exp(-\frac{t}{t_d}) \quad (14)$$

where $k_{\mathrm{m}}$, $t_{\mathrm{d}}$, $u_{\mathrm{sp}}$ is the half-saturation constant when the reaction rate is reduced by 50%, the time

constant and the maximum urease constant, respectively.

### *2.4 Calcium carbonate deposition and porosity evolution*

Calcium carbonate produced by biomineralization deposit between the sand particles, fill pores, reduce the porosity and permeability of the sand (Van Paassen et al., 2010; DeJong et al., 2010; Burbank et al., 2011; Jiang et al., 2017; Wang et al., 2019). Assuming that the deposited calcium carbonate does not transport in the reaction time, there are:

$$\frac{\partial \varphi}{\partial t} = -\frac{1}{\rho_c} m_{\mathrm{CaCO_3}} \varphi k_{rea} \tag{15}$$

$$\frac{\partial c_{CaCO_3}}{\partial t} = m_{CaCO_3} \varphi k_{rea} \tag{16}$$

where $\rho_c$ is the density of the calcium carbonate. In addition, the Kozeny-Carman (*KC*) equation and the effective porosity are adapted in this paper according to Wang et al. 2020a, in which the enough details can be obtained. The boundary conditions at multi-process are shown in Table 1.

**Table 1** Boundary conditions of the MICP process.

| **Multi-process** | **Phase B** | **Phase R** | **Phase C** |
|---|---|---|---|
| **Top** | | | |
| $q_1$ | $q_{in}$ | / | / |
| $p$ | / | $p_{atm}$ | $p_{atm}$ |
| $C_{bacl}$ | $C_{bact0}$ | $(\boldsymbol{D}^* \cdot \varphi \nabla C) \cdot \mathbf{n} = 0$ | / |
| $C_{urea}$ | / | / | $(\boldsymbol{D}^* \cdot \varphi \nabla C) \cdot \mathbf{n} = 0$ |
| $C_{Ca2+}$ | / | / | $(\boldsymbol{D}^* \cdot \varphi \nabla C) \cdot \mathbf{n} = 0$ |
| $C_{NH4+}$ | / | / | $(\boldsymbol{D}^* \cdot \varphi \nabla C) \cdot \mathbf{n} = 0$ |
| **Bottom** | | | |
| $q_2$ | / | / | $q_{in}$ |
| $p$ | $p_{atm}$ | $p_{atm}$ | / |
| $C_{bacl}$ | $(\boldsymbol{D}^* \cdot \varphi \nabla C) \cdot \mathbf{n} = 0$ | $(\boldsymbol{D}^* \cdot \varphi \nabla C) \cdot \mathbf{n} = 0$ | $(\boldsymbol{D}^* \cdot \varphi \nabla C) \cdot \mathbf{n} = 0$ |
| $C_{urea}$ | / | / | $C_{urea0}$ |
| $C_{Ca2+}$ | / | / | $C_{Ca2+0}$ |
| $C_{NH4+}$ | / | / | $C_{NH4+0}$ |

## 3. Results and analysis

### *3.1 The relationship between scale size and the longitudinal dispersivity*

Dispersivity is a key parameter in the convection-diffusion, which characterizes the dispersion characteristics of solutes, and affected by scale size. The dispersivity measured indoors is often different from that measured in the field by one or several orders of magnitude, thus the results of the indoor test cannot be applied to the field. Some studies have found that the dispersivity increase with the increase of the scale size. (Moradi et al., 2020; Zheng et al., 2015; Gelhar et al., 1992; Godoy et al., 2018; Wang et al., 2020b). According to this theory, the dispersivity obtained indoors can be extrapolated to the field scale by scale analysis. However, it is still a worldwide problem due to the uncertainties in scale size. Thus, a large amount of data between longitudinal dispersivity and scale size are summarized in Fig. 3 (Gelhar 1992; Fauriel et al., 2012; Zhao et al., 2021; Wang et al., 2020a; Boufadel et al., 2011; Landa-Marban et al., 2021; Nassar et al., 2018; van Wijingaarden et al., 2013). It can be found that the longitudinal dispersivity in saturated porous media increase with the increase of the scale size. The relationship is as follows:

$$lg\alpha_L = 0.88342 lgL_0 - 1.1267 \tag{17}$$

where $L_0$ represents the scale size along the main flow direction. When scale size increases logarithmically from $10^{-2}$ m to $10^5$ m, longitudinal dispersivity also shows a trend of increasing from $10^{-3}$ m to $10^4$ m.

According to Fig. 3, the dispersivity measured in the laboratory can be extended to field. Although it is not particularly accurate, it still has good reliability. The value of the longitudinal dispersivity at different scales can be calculated by Eq. 17, and then the simulation of the transport and reaction of the corresponding substances can be carried out. Especially for the large-scale MICP multi-process, there is no report on the influence of the scale size on the calculation results.

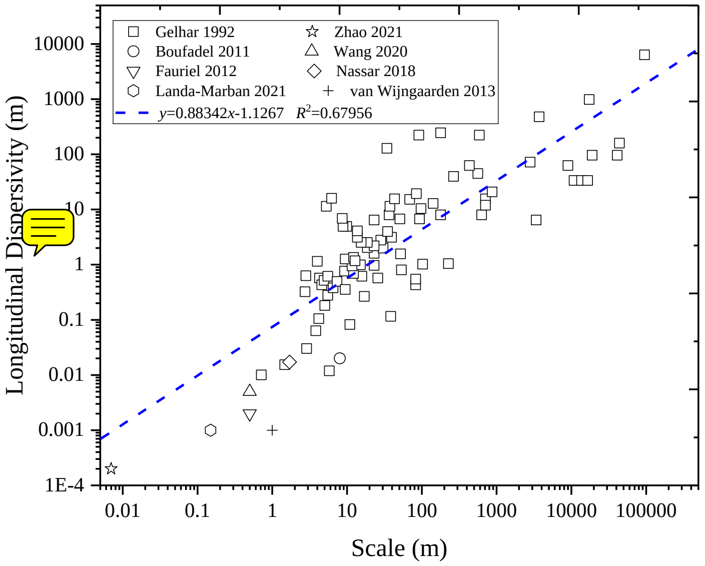


**Fig. 3** Longitudinal dispersivity versus of the scale size in saturated porous media.

### *3.2 Model calibration and verification*

The results of the Martinez (2013) are used to calibrate and verify the model. As shown in Table 2, Test 1B is used for calibrating. The parameters attachment rate $k_{att}$ and maximum urease constant $u_{sp}$ were determined according to Test 1B. And the Test 2B and Test 3A are used for verification. According to the Fig. 4(a), although the value of the penetration curve of the suspended bacteria is far from the measured value at the time of 1h, the numerical model can able to reflect the penetration law of the suspended bacteria. The calcium carbonate profile is in high agreement with the measured value, and the permeability coefficient after the MICP reaction is also similar. The permeability coefficient measured experimentally is $4.5\times10^{-4}$ cm/s, while the calculated value is $4.11\times10^{-4}$ cm/s. All calibration parameters are summarised in Table 3.

**Table 2** Treatment steps of calibration and verification case.

| **Test No.** | **Test1B(calibration)** | **Test2B(verification)** | **Test3A(verification)** |
|---|---|---|---|
| Sand length [illegible]m) | 50 | 50 | 50 |
| Initial porosity | 0.37 | 0.35 | 0.36 |
| Initial perimeability (cm/s) | 3e-2 | 3.5e-3 | 2.2e-2 |
| **PhaseB** | | | |
| Flow type | Continous | Continous | Continous |
| Injecting rate (mL/min) | 10 | 10 | 10 |

| | | | |
|---|---|---|---|
| Source | top | bottom | top |
| Initial cell concentration (cells/mL) | 7.2e5 | 2.5e5 | 4.3e5 |
| Volume of cell solution (mL) | 442.1 | 393.9 | 598.4 |
| **The Time of PhaseR (h)** | 8.3 | 12 | 8 |
| **PhaseC** | | | |
| Flow type | Continous | Continous | Stop |
| Injecting rate (mL/min) | 2.2 | 4.4 | 10 |
| Source | bottom | bottom | bottom |
| $C_{\text{urea}}:C_{\text{NH4+}}:C_{\text{Ca2+}}$ | 0.3:0.3:0.1 | 0.3:0.3:0.1 | 0.05:0.05:0.05 |
| Injecting time (h) | 81.267 | 48.769 | 2h/pulse |

**Table 3** Model parameters.

| **Model parameters** | **Value** | **Reference** |
|---|---|---|
| Water density at 25℃ $\rho_{\text{w}}$ ($kg/m^3$) | 1000 | |
| Viscosity at 25℃ $\mu_{\text{l}}$ ($Pa \cdot s$) | 0.001 | |
| Constant $a$ | 0.33 | Wang (2020) |
| Critical porosity $\varphi_c$ | 0.25 | estimated |
| Diffusion constant $\boldsymbol{D}$ ( $m^2/s$ ) in water phase | 2e-9 | Wang (2020) |
| Attachment rate $k_{\text{att}}$ ( $1/s$) | 1.52e-3 | estimated |
| Decay rate $k_{\text{d}}$ ($1/s$) | 3.18e-7 | Wang (2020) |
| Half saturation constant $k_{\text{m}}$ ($mol/m^3$) | 10 | Van Paassen(2009) |
| Time constant $t_{\text{d}}$ ($s$) | 288000 | Fauriel(2012) |
| Maximum urease constant $u_{\text{sp}}$ ($mol/m^3/s/cells/mL$) | 1.4e-8 | estimated |
| Molar mass $m_{\text{urea}}$, $m_{\text{Ca2+}}$, $m_{\text{NH4+}}$, $m_{\text{CaCO3}}$ ($kg/mol$) | 0.078,0.04,0.018,0.1 | |
| Calcaite density $\rho_{\text{c}}$ ($kg/m^3$) | 2710 | |

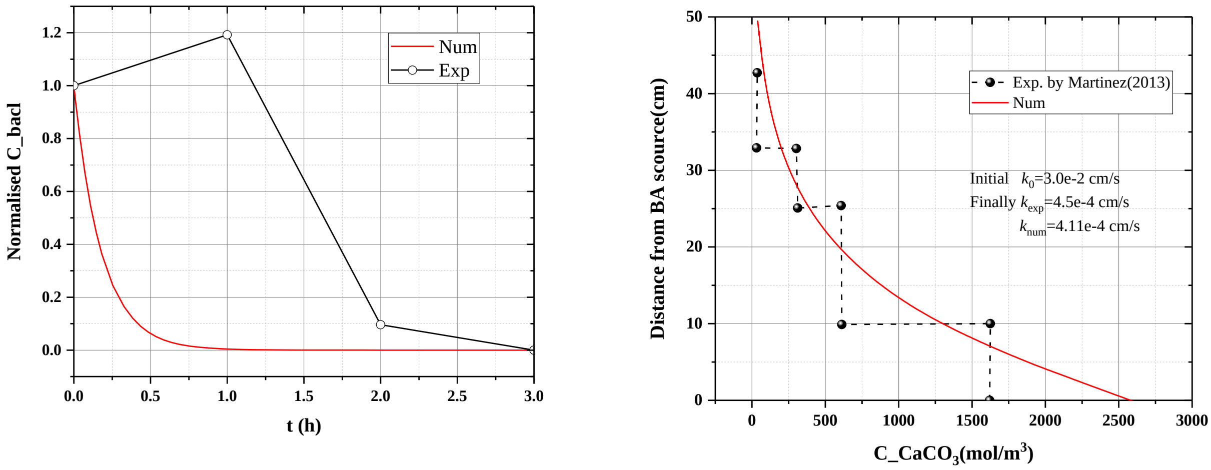


(a) (b)

**Fig. 4** The results of the parameter calibration: (a) Penetration curves of the suspended bacteria; (b) Axial profile of the measured carbonate and the numerical carbonate of Test1B.

The verification results are shown in Fig. 5. It can be found that when considering the size effect, the concentration of attached bacteria is higher than that without considering the size effect (Fig. 5(a)), leading to a higher calcium carbonate content, which is more in line with the calcium carbonate content measured in the experiment (Fig. 5(b)). However, it has little effect on the penetration law of the suspended bacteria (Fig. 5(c~d)). From Fig. 5(e), the concentration of the suspend bacteria is higher than that without considering the size effect in Test3A, the distribution characteristics of the suspend bacteria are close to the measured values when the size effect is considered. Fig. 5(f) shows the evolution of the urea, calcium and ammonium over the time in Test 3A. After considering the size effect, the consumption of reactants (urea and calcium) at the same time is larger than that in the case which the size effect is not considered. Correspondingly, the product (ammonium) is also higher.

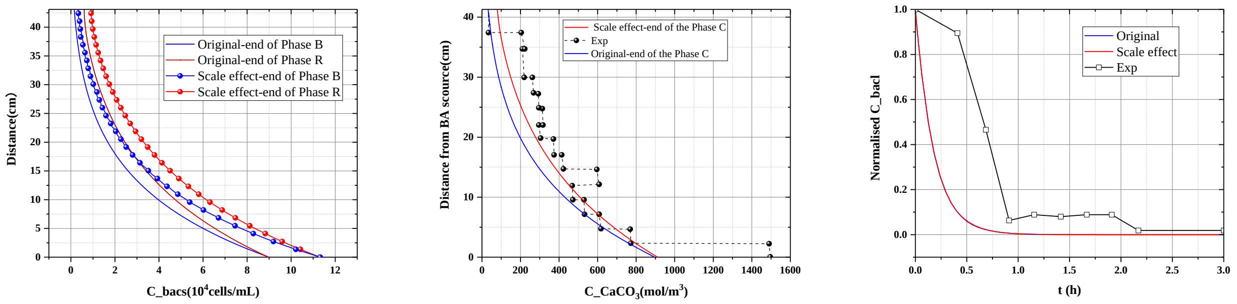


(a) (b) (c)

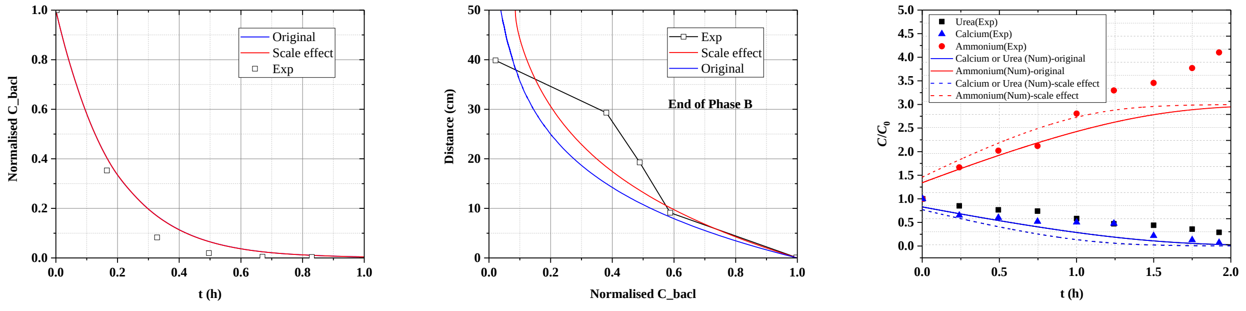


(d) (e) (f)

**Fig. 5** Axial profile of (a) the attached bacteria in Test2B; (b) the carbonate in Test2B; (c) Penetration curves of the suspended bacteria in Test2B; (d) Axial profile of the suspended bacteria in Test3A; (e) Penetration curves of the suspended bacteria Test3A; (f) Evolution of the urea, calcium and ammonium over time in Test3A.

In summary, this numerical model can able to reflect the kinetics of biomineralization and the law of calcium carbonate precipitation, it is notes that the calculated results are closer to the experimental data after taking the scale size into account. Table 4 shows the design of calculation cases, the effects of scale size $L_0$, bacterial injecting rate $q_1$, and the initial porosity $\varphi_0$ on the MICP multi-process were studied systematically.

**Table 4** Design of calculation cases.

| Variables | Value | Unit |
|---|---|---|
| Initial bacterial concentration $C_{bact0}$ | 7.2e5 | cells/mL |
| Injecting rate of cell solution $q_1$ | 5,**10**,15,20,25 | mL/min |
| Injecting volume of the cell solution $V_c$ | 442.1 | mL |
| Initial perimeability coefficient $k_0$ | 3e-4 | m/s |
| Initila porosity $\varphi_0$ | 0.35,**0.37**,0.39,0.41,0.43 | |
| Scale size $L_0$ | 0.1,**0.5**,1,5,10 | m |
| The time interval of the PhaseR | 8.3 | h |
| $C_{urea}$:$C_{NH4+}$:$C_{Ca2+}$ | 0.3:0.3:0.1 | mol/L |
| Injecting rate of cementing solution $q_2$ | 2.2 | mL/min |
| Injecting time of cementing solution $t_2$-$t_1$ | 81.267 | h |

The parameters of the standard calculated group are bold.

### *3.3 Effect of the scale size*

Figs. 6(a~c) show the axial profile of the suspended bacteria with the different scale size at the end of the Phase B. After considering the scale size, the suspended bacteria are higher than that without

considering the scale size. The point where the suspended bacteria is zero is defined as the critical point (Fig. 6(b)), which indicates that the penetration ability of the substance. In order to quantify the effect of the scale size, define the error of the mean $\delta$ (%):

$$\delta = \frac{C_{i(scale\ effect)} - C_{i(original)}}{C_{i(original)}} \times 100\% \quad (18)$$

where $i$ include the suspended bacteria, the attached bacteria, urea, calcium, ammonium and carbonate. As shown in Fig. 6(d), the mean error of the average suspended bacteria increases with the increase of the scale size. Figs. 7(a~c) show that the attached bacteria also showed similar laws to the suspended bacteria.

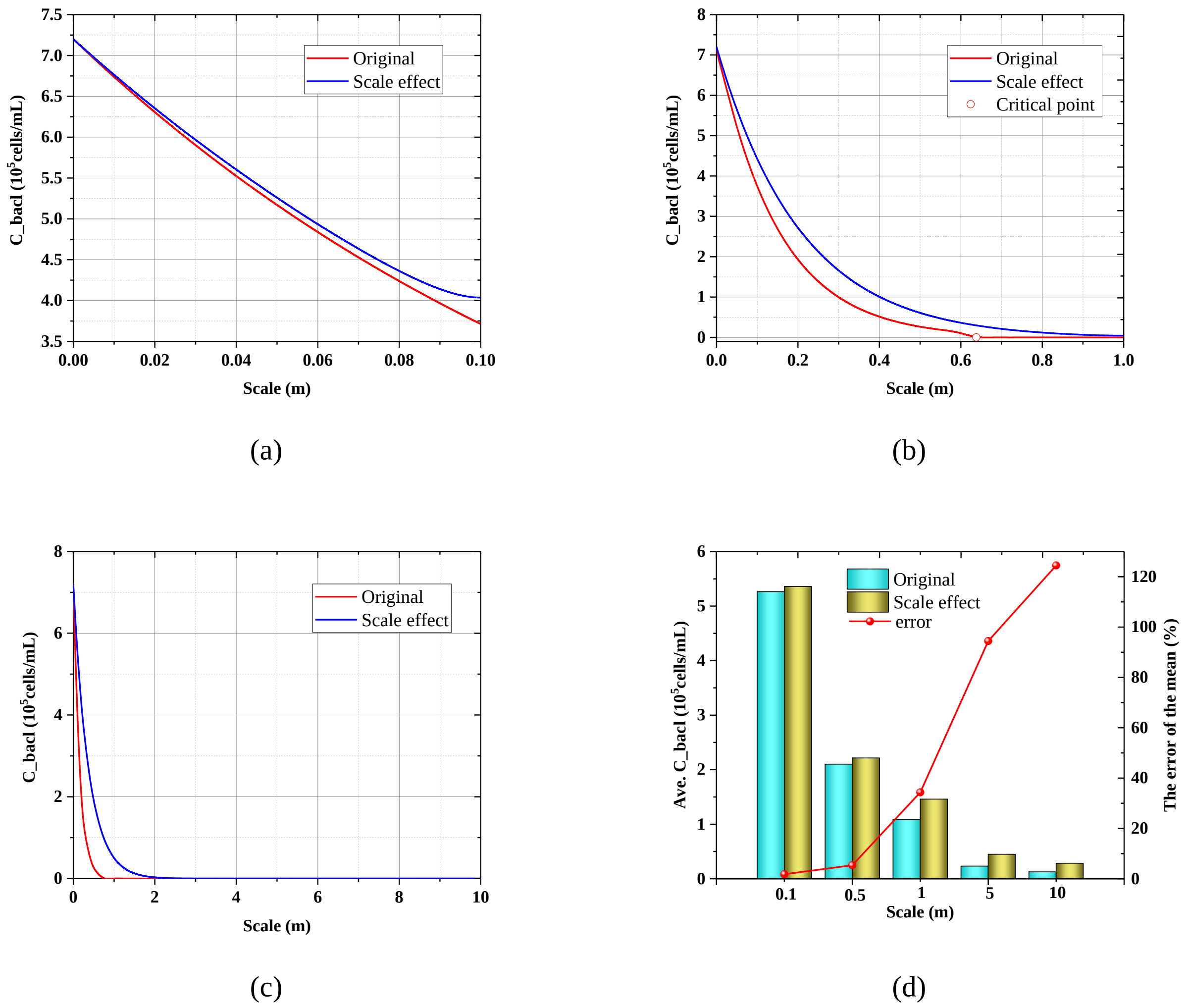


**Fig. 6** Axial profile of (a) the suspended bacteria in 0.1m; (b) the suspended bacteria in 1.0m; (c) the suspended bacteria in 10m at the end of the PhaseB; (d) The influence of the scale size on the average suspended bacteria.

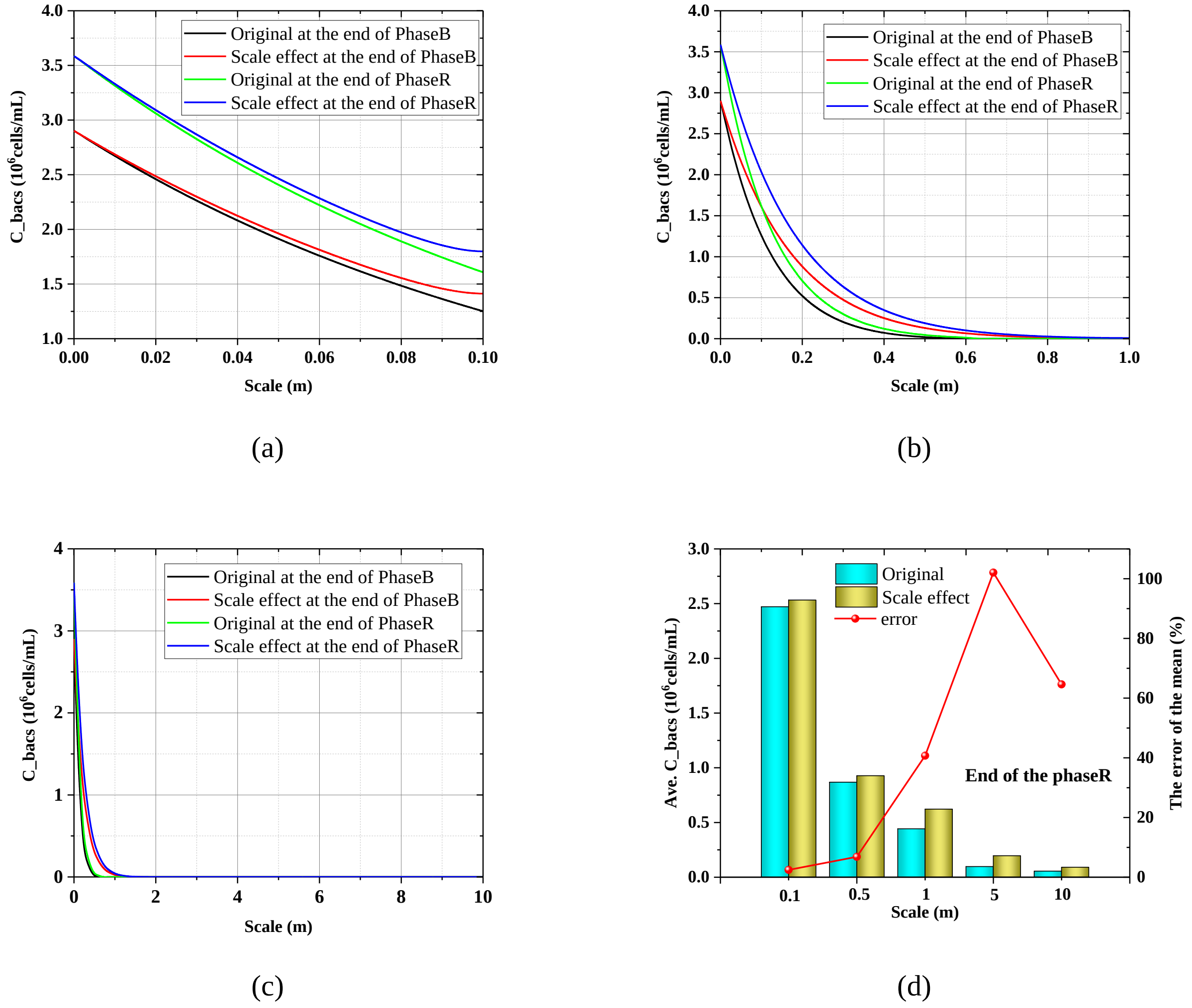


**Fig. 7** Axial profile of (a) the attached bacteria in 0.1m; (b) the attached bacteria in 1.0m; (c) the attached bacteria in 10m at the end of the PhaseB; (d) The influence of the scale size on the average attached bacteria.

Fig. 8 shows the axial profile of the concentration of the urea (calcium) and the ammonium at the end of the Phase C, when the sand column height is 0.1 m, considering the scale size has little effect on the concentration of urea and ammonium. When the sand column height increases to 1 m, more urea is consumed after considering the size effect, resulting in more ammonium ions. The influence after considering the scale size is more significant at the 10 m sand column height.

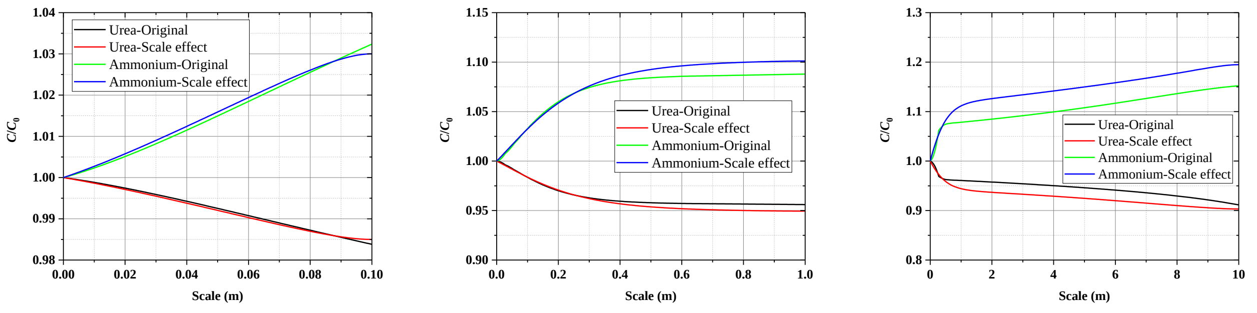


(a) (b) (c)

**Fig. 8** Axial profile of the urea and ammonium in (a) 0.1m; (b) 1.0m; (c) 10m at the end of the PhaseC.

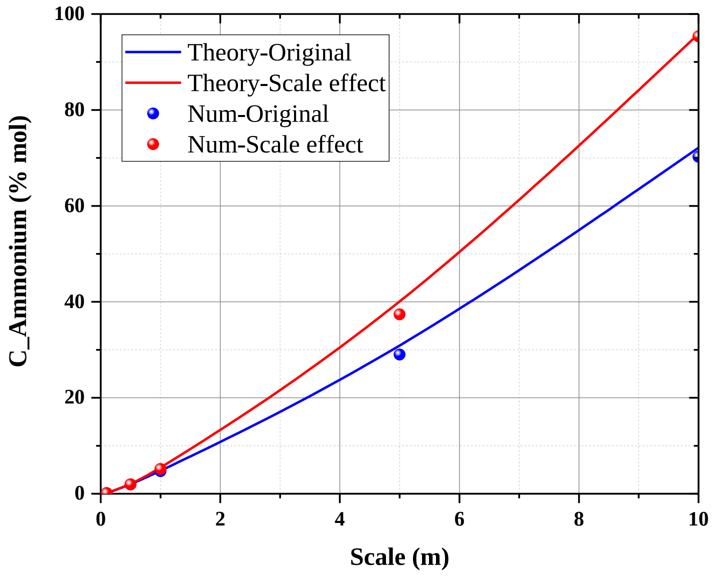


**Fig. 9** The comparison between the theoretical ammonium production calculated by 100% conversion rate and the numerical ammonium production.

Suppose the conversion rate is 100%, the theoretical ammonium production (consumption of 1 mol urea to produce 2 mol ammonium) is highly consistent with the numerical ammonium production. And with the increase of scale size, the difference between the ammonium concentration after considering the size effect and the ammonium concentration without considering the size effect increases sequentially (Fig. 9). The maximum value of the difference can be as high as 28% when the calculated size is 10 m in this paper.

Carbonate is the product of the MICP multi-process, which increases strength, stiffness, and reduces permeability and porosity of the sand. Figs. 10(a~c) show the axial profile of the carbonate with the different scale size, and Fig. 10(d) gives the average carbonate value and the error. The effect of the

scale size on carbonate is also similar to that of the suspended bacteria.

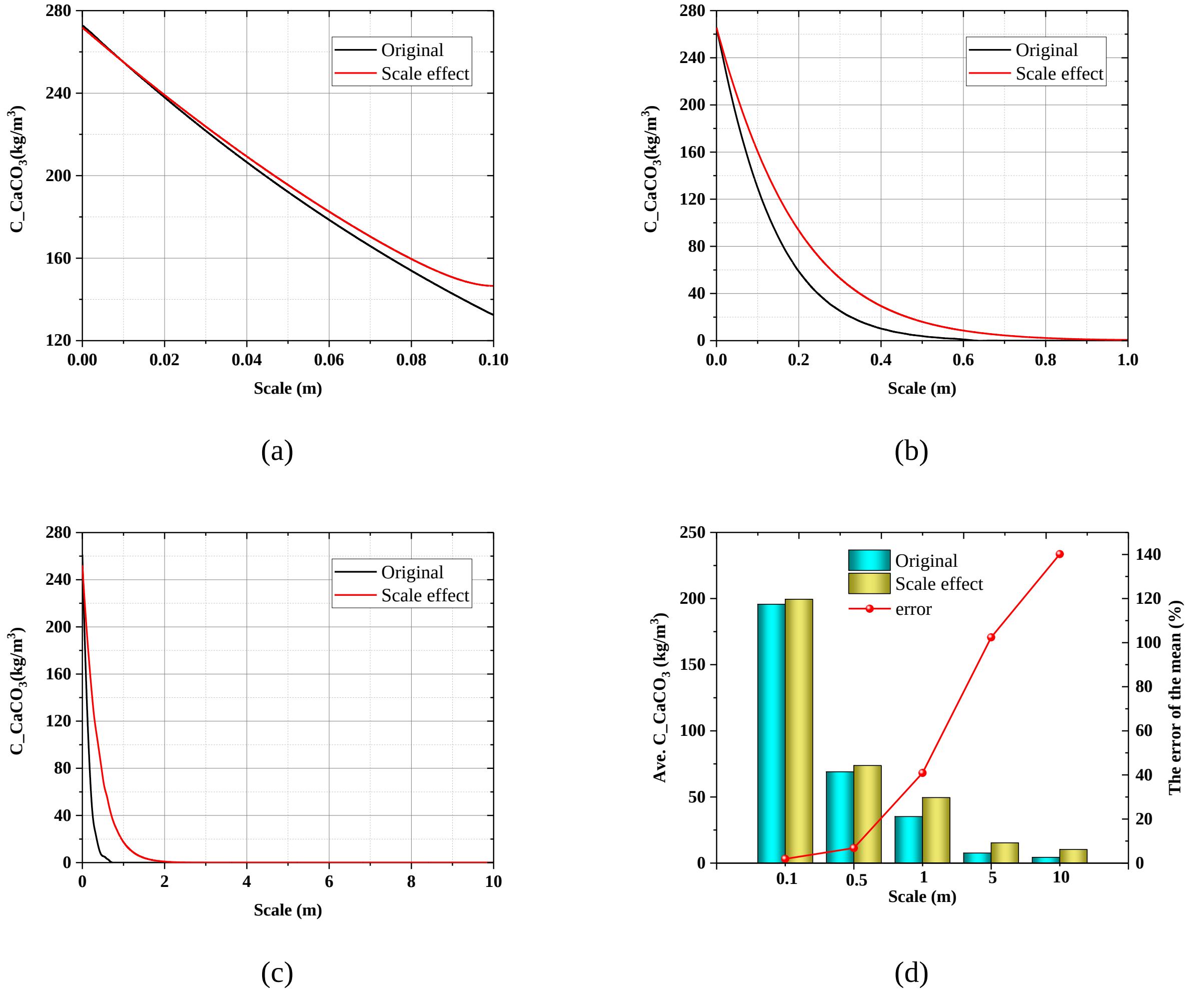


**Fig.10** Axial profile of the carbonate in (a) 0.1m; (b) 1.0m; (c) 10m at the end of the PhaseB; (d) The influence of the scale size on the average carbonate.

Table 5 summarized the location of the critical point of each substance in the MICP multi-process. When the scale size is less than 0.5m, all substances penetrate the entire calculation height (the penetration ratio is 100%). At the end of the Phase B, the penetration ratio of the $C_{bacl}$ for 0.1 m, 0.5 m, 1m, 5 m, 10 m scale size is 100%, 100%, 63.8%, 18.66%, 15.87% without considering size effect. While the value of considering the size effect is 100%, 100%, 100%, 60.7%, 39.68%, respectively. This suggests that ignoring the size effect underestimates penetration capability, which is detrimental to model accuracy and cost considerations. Both the penetration ratio of the $C_{bacs}$ at the end of Phase B and the carbonate at the end of the Phase C show a similar pattern of the $C_{bacl}$.

**Table 5** Influence of the scale on the critical point location.

| Scale size (m) | 0.1 | 0.5 | 1 | 5 | 10 |
|---|---|---|---|---|---|

| | | | | | |
|---|---|---|---|---|---|
| | | $C_{bacl}$ | | | |
| Original-end of PhaseB | /*100 | /*100 | 0.638/*63.8 | 0.933/*18.66 | 1.587/*15.87 |
| Scale effect-end of PhaseB | /*100 | /*100 | /*100 | 3.035/*60.7 | 3.968/*39.68 |
| | | $C_{bacs}$ | | | |
| Original-end of PhaseB | /*100 | /*100 | 0.619/*61.9 | 0.654/*13.08 | 0.675/*6.75 |
| Scale effect-end of PhaseB | /*100 | /*100 | /*100 | 2.936/*58.72 | 2.579/*25.79 |
| Original-end of PhaseR | /*100 | /*100 | 0.631/*63.1 | 0.694/*13.88 | 0.754/*7.54 |
| Scale effect-end of PhaseR | /*100 | /*100 | /*100 | 3.115/*62.3 | 2.698/*26.98 |
| | | $C_{CaCO3}$ | | | |
| Original-end of PhaseC | /*100 | /*100 | 0.631/*63.1 | 0.694/*13.88 | 0.754/*7.54 |
| Scale effect-end of PhaseC | /*100 | /*100 | /*100 | 1.448/*28.96 | 1.984/*19.84 |

*The penetration ratio, defined as a percentage of the ratio of the critical point location to the total height.

### *3.4 Effect of the porosity and the bacteria injecting rate*

The porosity affects the convection diffusion reaction of the solute, so it is also necessary to pay attention to its effect on the MICP multi-process. Here, a 0.5 m-long model is used to conduct a study. From Figs. 11(a~b), under the condition of small initial porosity, there are more suspended and attached bacteria, which relies on the sieving effect of the sand on bacteria. But the initial porosity has no effect on the maximum concentrations of the suspended and attached bacteria. It can be found from Fig. 11(c) that small porosity is more conducive to the uniform distribution of carbonate. The initial porosity has less effect on the average carbonate. But the maximum carbonate content, which controls the permeability of the porous media, increases with the initial porosity (Fig. 11(d)). The value of which is 251, 266, 280, 294, 309 kg/m$^3$ for the initial porosity of 0.35, 0.37, 0.39, 0.41, 0.43.

As shown in Fig. 12(a), the concentration and penetrating ability of the suspended bacteria increase with the increase of the bacteria injecting rate at the same injecting time. From Fig. 12(b), when the injecting rate is 5 mL/min, the attached bacteria still had a critical point in the height of 0.5 m, which indicated that the attached bacteria does not completely penetrate the sand column at this flow rate. When the injecting rate increase to 10 mL/min, the critical point of the attached bacteria disappears. And the concentration of the attached bacteria increases with the increase of the injecting rate. Fig. 12(c) shows the spatial distribution of the carbonate with different injecting rates, which increase sequentially with the increase of the injecting rates. This also shows that the uniformity of cementation also improves with the increase of the injection rate. When the injection rate is less than the reaction

rate, carbonate is rapidly deposit and then plugs the pores, so that no carbonate precipitate far from the grouting port. When the injection rate is greater than the reaction rate, the solute fills the entire sand column first and then starts to react to form a more uniform carbonate distribution. Fig. 12(c) summarized the effect of injecting rate on the suspended bacteria, the attached bacteria, the average carbonate, and the maximum carbonate. With the increase of injecting rate, all the average suspended bacteria, the average attached bacteria and the average carbonate show a significant increasing trend. And it has a more uniform distribution of the calcium carbonate at higher injecting rates, which is very beneficial for ground improvement and load transfer. While the injecting rate has little effect on the maximum carbonate content, the location of which is near the grouting port.

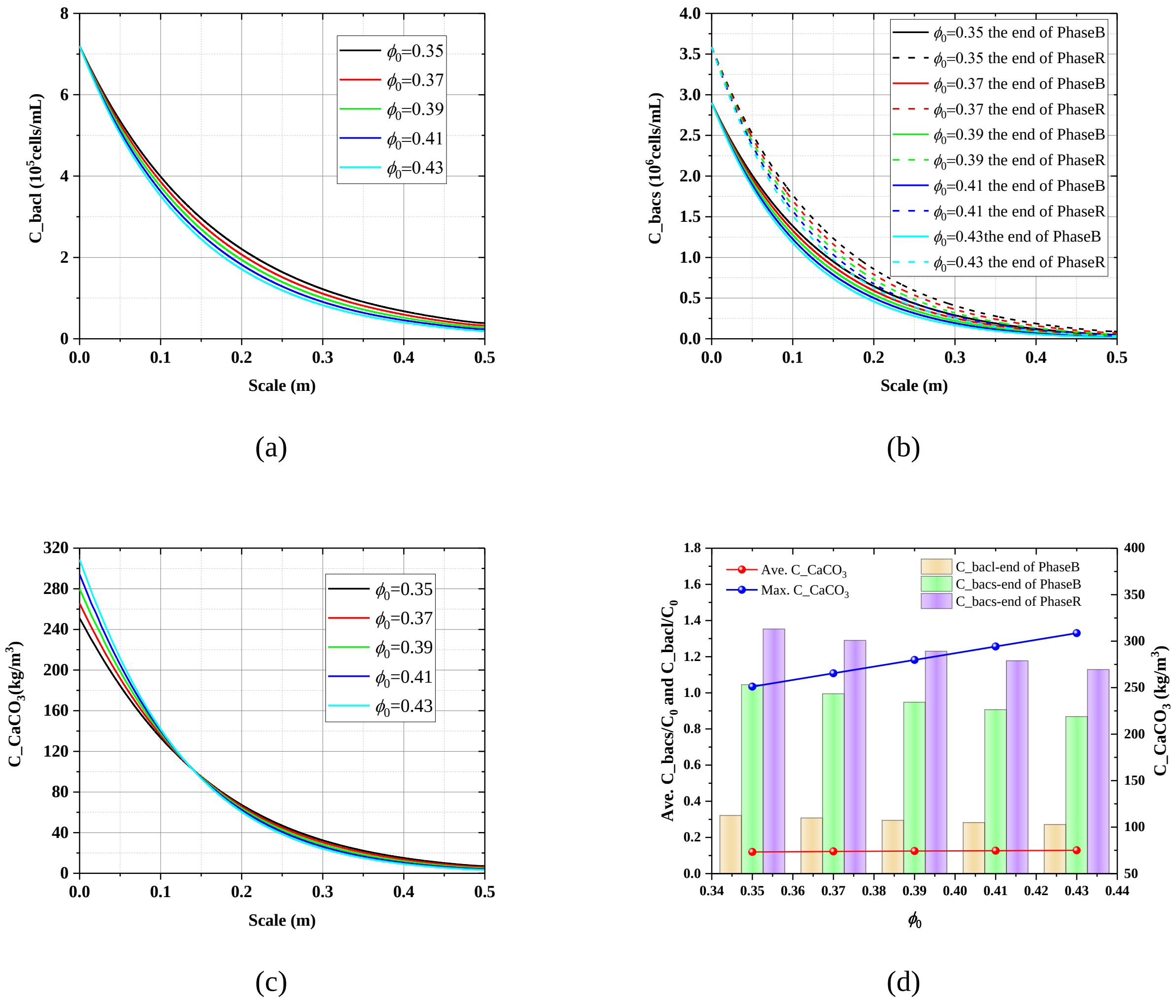


**Fig. 11** The influence of the initial porosity on the (a) axial profile of the suspended bacteria at the end of PhaseB; (b) axial profile of the attached bacteria at the end of the PhaseB and PhaseC; (c) axial profile of the carbonate

at the end of the PhaseC; (d) the average bacteria and the carbonate.

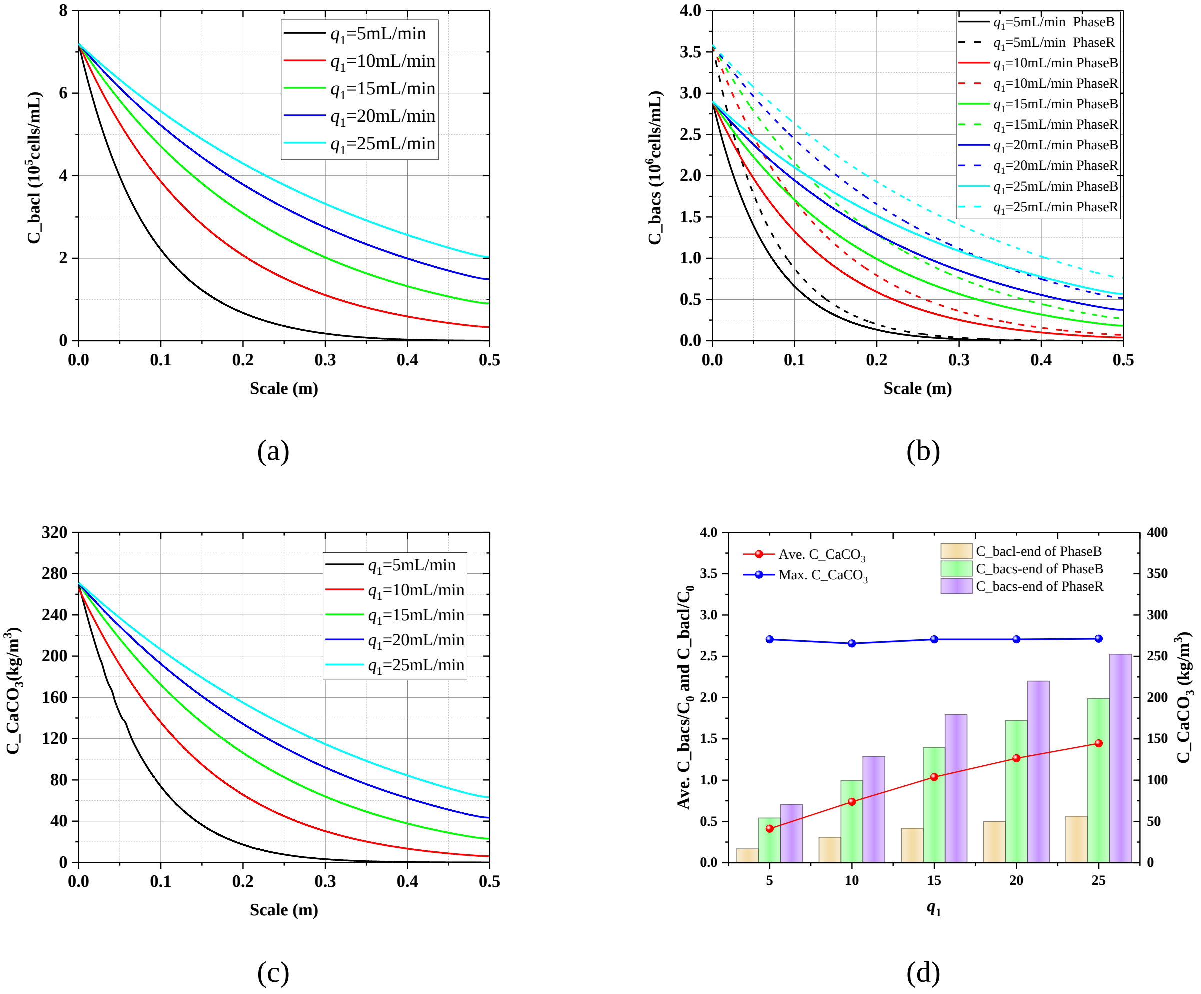


**Fig. 12** The influence of the bacteria injecting rate on the (a) axial profile of the suspended bacteria at the end of PhaseB; (b) axial profile of the attached bacteria at the end of the PhaseB and PhaseC; (c) axial profile of the carbonate at the end of the PhaseC; (d) the average bacteria and the carbonate.

## 4. Applicability and process of the numerical model

Based on this numerical model, the temporal and spatial evolution of various substance (urea, calcium, ammonium, bacteria) concentrations, the porosity and permeability of the ground can be obtained in real time, and more importantly, the influence of the scale size on the simulated results is considered. Different from other MICP numerical models with many parameters, this model can simulate MICP multi-processes with different sand types when three parameters (the constant attachment rate $k_{att}$, the maximum urease constant $u_{sp}$, critical porosity $\varphi_c$) are calibrated. Reliable numerical calculation can provide an important reference for the selection of on-site construction plans

and parameters. As shown in Fig. 13, when MICP technology is used to reinforce the ground, slopes and banks, it mainly includes the following steps: 1) The basic parameters (porosity, permeability coefficient, scale size) are determined by means of in-situ testing; 2) In order to obtain the optimal parameters (bacterial concentration, injecting rate, injecting time, cement solution concentration), the sensitivity analysis based on this theoretical model is necessary. Uniform calcium carbonate distribution is beneficial for load transfer and durability. 3) On-site reinforcement treatment is carried out according to the optimal parameter combination, the strength, stiffness and the uniformity of the ground are evaluated by shear wave velocity, electrical conductivity, drilling sampling and other methods. If the engineering requirements are not met, the secondary reinforcement treatment shall be carried out.

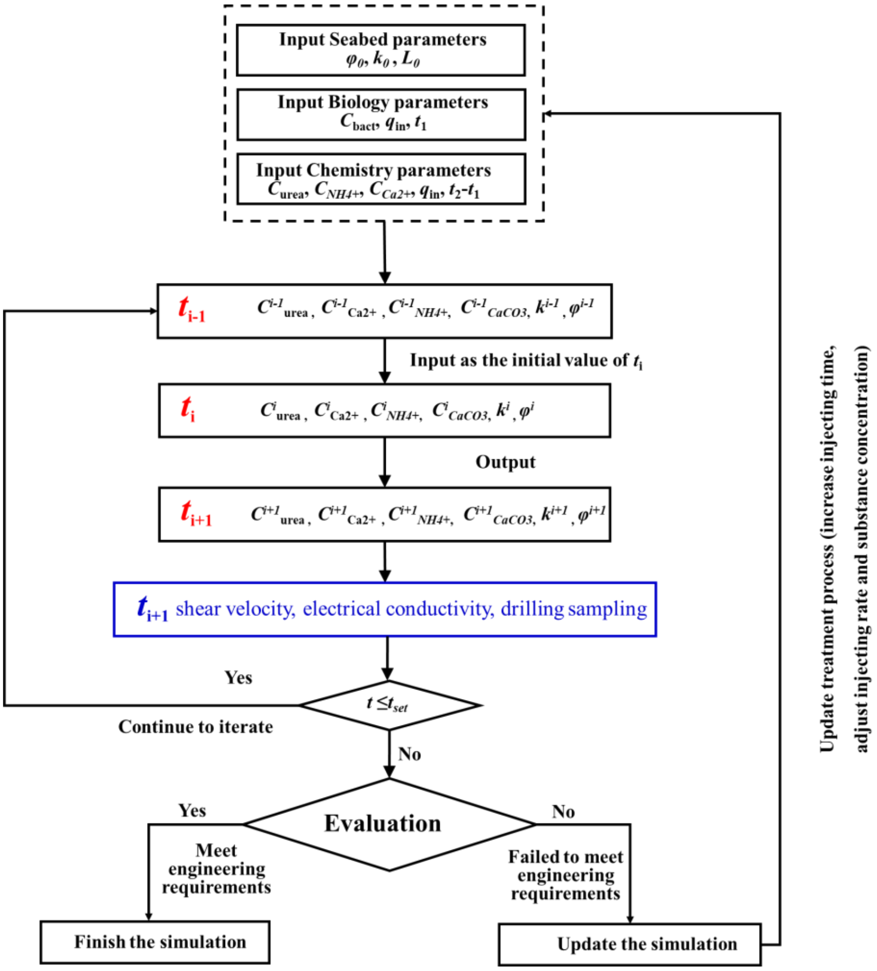


**Fig. 13** MICP field reinforcement operation process and numerical simulation.

## 5. Conclusions

In this paper, the preliminary relationship between scale size and the dispersivity was established, and then the numerical model of the MICP multi-process was optimized. Moreover, the influences of the size effect, initial porosity and the bacterial injecting rate were also analysed.

1) The relationship between the dispersivity and the scale size presented as a substantially linear relationship in the double logarithmic coordinate system. Considering the size effect is closer to the experimentally measured value. According to this relationship, the convection diffusion reaction in the MICP multi-process can be preliminarily simulated.
2) After considering the size effect, the suspended and attached bacteria are higher than that without considering the size effect, which leading to a higher calcium carbonate content. Scale has little effect on the penetration law of the suspended bacteria. The mean error of the suspended bacteria and the average carbonate content increases with the increase of the scale size.
3) The bacterial injecting rate and initial porosity have significant effects on the distribution of the suspended bacteria, the attached bacteria and the carbonate distribution. The maximum carbonate content increases with the increase of the initial porosity, and the average carbonate showed a significant increasing trend with the increasing of the bacterial injecting rate. And it has a more uniform distribution of the calcium carbonate at higher injecting rates or smaller initial porosity, which is very beneficial for ground improvement and load transfer. Both the smaller initial porosity and higher bacterial injecting rate are more conducive to the uniform distribution of carbonate.
4) It is recommended to consider the influence of the scale size on the MICP multi-process in the simulation of the microbial mineralization kinetic model.

## Data Availability Statement

All data used to support the findings of this study are included within the article.

## Defining the Role of Authors and Contributors

**Zhu Xianxian**: Conception, Writing-original draft, Investigation.

**Wang Jianhua**: Conception, Methodology, Investigation.

**Wang Haili**: Conception, Writing-review & editing

**Yujie Li**: Conceptualization, Investigation, Methodology, Writing-original draft

## Conflicts of Interest

The authors declare that there is no conflict of interest regarding the publication of this paper.


## Acknowledgements

The authors would like to acknowledge the supports by the Zhejiang Water Conservancy Key Science and Technology Project (RB2120), Hainan Special PhD Scientific Research Foundation of Sanya Yazhou Bay Science and Technology City (HSPHDSRF-2022-04-002).


## References


Alexandra, C.S., Stuart, K.H., Jorat, M.E., 2021. Flume study on the effects of microbial induced calcium carbonate precipitation (MICP) on the erosional behaviour of fine sand. *Géotechnique* 71(12), 1135-1149.

Bear J. 1972. Dynamics of Fluids in Porous Media. American Elsevier, New York, NY, 764pp.

Boufadel, M.C., Xia, Y.Q., Li, H.L., 2011. Modeling solute transport and transient seepage in a laboratory beach under tidal influence. *Environmental Modelling & Software* 26, 899-912.

Burbank, M.B., Weaver, T.J., Green, T.L., et al., 2011. Precipitation of carbonate by indigenous microorganisms to strengthen liquefiable sand. *Geomicrobiology Journal* 28(4), 301-312.

Bu, C., Lu, X., Zhu, D., et al., 2022. Soil improvement by microbially induced calcite precipitation (MICP): a review about mineralization mechanism, factors, and soil properties. *Arabian Journal of Geosciences*, 15(9): 1-25.

DeJong, J.T., Mortensen, B.M., Martinez, B.C., et al., 2010. Bio-mediated sand improvement. *Ecological Engineering* 36, 197-210.

Fang, L.Y., Niu, Q.J., Cheng, L., et al., 2021. Ca-mediated alleviation of $Cd^{2+}$ induced toxicity and improved $Cd^{2+}$ biomineralization by *Sporosarcina pasteurii*. *Science of The Total Environment* 787(15), 147627.

Fauriel, S., & Laloui, L., 2012. A bio-chemo-hydro-mechanical model for microbially induced carbonate precipitation in sands. *Computers and Geotechnics* 46, 104-120.

Gelhar, L.W., 1992. A critical review of data on field-scale dispersion in aquifers. *Water Resources Research* 28(7), 1955-1974.

Godoy, V.A., Zuquette, L.V., Gomez-Hernandez, J.J., 2018. Size effect on hydraulic conductivity and solute transport: Small and large-scale laboratory experiments and field experiments. *Engineering geology* 243(4), 196-205.

Hommel, J., Lauchnor, E., Phillips, A., et al., 2015. A revised model for microbially induced carbonate

precipitation: Improvements and new insights based on recent experiments. *Water Resources Research* 51, 3695-3715.

Huang W., Mou Y., Li Y., et al., 2022. Study on durability of MICP treated cohesive soils under dry–wet cycle and freeze–thaw cycle. *Arabian Journal of Geosciences*, 15(5): 1-10.

Jiang, N. J., & Soga, K., 2017. The applicability of microbially induced carbonate precipitation (MICP) for internal erosion control in gravel-sand mixtures. *Géotechnique* 67(1), 42-55.

Kashizadeh, E., Mukherjee, A., Tordesillas, A., 2021. Experimental and numerical investigations on confined granular systems stabilized by bacterial cementation. *International Journal of Geomechanics* 21(1): 04020244.

Kou, H., Wu, C., Ni, P., et al., 2020. Assessment of erosion resistance of biocemented sandy slope subjected to wave actions. *Applied Ocean Research* 105, 102401.

Landa-Marban, D., Tveit, S., Kumar, K., Gasda, S.E., 2021. Practical approaches to study microbially induced carbonate precipitation at the field scale. *International Journal of Greenhouse Gas Control* 106, 103256.

Li, Y.J., Guo, Z., Wang, L.Z., et al., 2020. Interface shear behaviour between MICP-treated calcareous sand and steel. *Journal of materials in civil engineering* 33(2), 04020455.

Li, Y.J., Guo, Z., Wang, L., et al., 2022. An Innovative Eco-friendly Method for Scour Protection around Monopile Foundation. *Applied Ocean Research* 123, 103177.

Li, Y., Guo, Z., Wang, L., & Yang, H. (2023). A coupled bio-chemo-hydro-wave model and multi-stages for MICP in the seabed. *Ocean Engineering*, 280, 114667.

Lyu, C.H., Qin, Y.J., Tian, C., et al., 2022. Microbial induced carbonate precipitation contributes to the fates of Cd and Se in Cd-contaminated seleniferous sands. *Journal of Hazardous Materials* 423, 126977.

Minto, J.M., Lunn, R.J., Mountassir, G.E., 2019. Development of a Reactive Transport Model for Field-Scale Simulation of Microbially Induced Carbonate Precipitation. *Water Resources Research* 55(8), 7229-7245.

Moradi, G., Mehdinejadiani, B., 2020. An experimental study on scale dependency of fractional dispersion coefficient. *Arabian Journal of Geosciences* 13, 409.

Martinez, B.C., Dejong, J.T., Ginn, T.R., et al., 2013. Experimental optimization of microbial induced carbonate precipitation for sand improvement. *Journal of Geotechnical and Geo-environmental Engineering* 139(4), 587-598.

Mujah, D., Cheng, L., & Shahin, M.A. 2019. Microstructural and Geomechanical Study on Biocemented Sand for Optimization of MICP Process. *Journal of Materials in Civil Engineering* 31(4), 04019025.

Mohamed, K.N., Deviyani, G., Mehrdad, B., et al., 2017. Large-scale experiments in Microbially induced carbonate precipitation (MICP): reaction transport model development and prediction. *Water Resources Research* 54, 480-500.

Nishimura, I., & Matsubara, H., 2021. Coupling simulation of microbially induced carbonate precipitation and bacterial growth using reaction-diffusion and homogenisation systems. *Acta geotechnica* 16, 1315-1330.

Nassar, M.K., Gurung, D., Bastani, M., et al., 2018. Large-scale experiments in microbially induced carbonate precipitation (MICP): Reactive transport model development and prediction. *Water*

*Resources Research* 54, 480–500.

Song, M.H., Ju, T.Y., Meng, Y., et al., 2022. A review on the applications of microbially induced calcium carbonate precipitation in solid waste treatment and sand remediation. *Chemosphere* 290, 133229.

Salifu, E., MacLachlan, E., Iyer, K.R., et al., 2016. Application of microbially induced carbonate precipitation in erosion mitigation and stabilisation of sandy sand foreshore slopes: A preliminary investigation. *Engineering Geology* 201, 96-105.

Toride, N., Inoue, M., Leij, F., 2003. Hydrodynamic Dispersion in an Unsaturated Dune Sand. *Sand Science Society of America Journal* 67(3), 703-712.

Tao, J., Li, J., Wang, X., et al. Nature-inspired bridge scour countermeasures: Streamlining and biocementation. *Journal of Testing and Evaluation* 2018, 46(4): 1376-1390.

Wang, Q.R., Gu, H.C., Zhan, H.B., et al., 2020b. Mixing effect on reactive transport in a column with scale dependent dispersion. *Journal of Hydrology* 582, 124494.

Wang, X.R., Nackenhorst, U. 2020a. A coupled bio-chemo-hydraulic model to predict porosity and permeability reduction during microbially induced carbonate precipitation. *Advanced in Water Resources* 140, 103563.

Wang, Y., Soga, K., Dejong, J.T., et al., 2019. A microfluidic chip and its use in characterising the particle-scale behaviour of microbial-induced calcium carbonate precipitation (MICP). *Géotechnique* 69(12): 1086-1094.

Whiffin, V.S., 2004. Microbial $CaCO_3$ precipitation for the production of Biocement. Ph.D. Dissertation, Morduch University, West Australia, pp162.

Wang, Y.Z., Soga, K., Dejong, J.T., Kabla, A.J., 2019. A microfluidic chip and its use in characterising the particle-scale behaviour of microbial-induced calcium carbonate precipitation (MICP). *Géotechnique* 69(12), 1086-1094.

Whiffin, V.S., van-Paassen, L.A., & Harkes, M.P., 2007. Microbial carbonate precipitation as a sand improvement technique. *Geomicrobiology Journal* 24(5), 417-423.

Von Wolff, L., Weinhardt, F., Class, H. et al., 2021. Investigation of Crystal Growth in Enzymatically Induced Carbonate Precipitation by Micro-Fluidic Experimental Methods and Comparison with Mathematical Modeling. *Transport in Porous Media* 137, 327-343.

Van Wijingaarden, W.K., Vermolen, F.J., van Meurs, G.A.M., & Vuik, C., 2011. Modelling biogrout: a new ground improvement method based on microbially-induced carbonate precipitation. *Transport in Porous Media* 87, 397-420.

Van Wijingaarden, W.K., Vermolen, F.J., van Meurs, G.A.M., & Vuik, C., 2013. A mathematical model for Biogrout. *Computational Geosciences* 17, 463-478.

Van Paasen, L.A., 2009. Biogrout: ground improvement by microbially induced carbonate precipitation. Ph.D. Dissertation, Delft University of Technology, Delft, Netherlands.

Van Paassen, L. A., 2011. Bio-mediated ground improvement: From laboratory experiment to pilot applications. Geo-Frontiers: Advances in Geotechnical Engineering, 4099-4108. Dallas: ASCE.

Van Paassen, L.A., Ghose, R., Van der Linden, T.J.M., van der Star Wouter, R.L., & van Loosdrecht, M.C.M., 2010. Quantifying Bio-mediated Ground Improvement by Ureolysis: Large-Scale Bio-grout Experiment. *Journal of Geotechnical and Geo-environmental Engineering* 136(12), 1721-1728.

Xiao, P., Liu, H., Xiao, Y., et al., 2018. Liquefaction resistance of bio-cemented calcareous sand. *Sand*

*Dynamics and Earthquake Engineering*, 107: 9-19.

Xiao, P., Liu, H., Stuedlein, A.W., et al., 2019. Effect of relative density and biocementation on cyclic response of calcareous sand. *Canadian Geotechnical Journal* 56(12), 1849-1862.

Xiao, Y., Stuedlein, A.W., Pan, Z., et al., 2020. Toe-bearing capacity of precast concrete piles through biogrouting improvement. *Journal of Geotechnical and Geoenvironmental Engineering*, 146(12).

Xiao, Y., Ma, G., Wu, H., et al., 2022. Rainfall-induced erosion of biocemented graded slopes. *International Journal of Geomechanics* 22(1): 04021256.

Xiao, Y., Stuedlein, A.W., He, X., et al., 2021. Lateral responses of a model pile in biocemented sand. *International Journal of Geomechanics* 21(11): 06021027.

Yu, X., Rong, H., 2022. Seawater based MICP cements two/one-phase cemented sand blocks. *Applied Ocean Research* 118, 102972.

Zeng, C., Veenis, Y., Hall, C.A., Young, E.S., van der Star, W.R.L., Zheng, J.J., & van Paassen, L.A., 2021. Experimental and numerical analysis of a field trial application on microbially induced carbonate precipitation for ground stabilization. *Journal of Geotechnical and Geo-environmental Engineering* 147(7), 05021003.

Zeng, F., Gao, Y.W., Sun, Y.Y., Shi, X.Q., Xu, H.X., Wu, J.C., 2015. Influence of flow velocity and spatial heterogeneity on DNAPL migration in porous media: insights from laboratory experiments and numerical modelling. *Hydrogeology Journal* 23, 1703-1718.

Zhao, C., He, X., Hu, R., Liu, H.L., et al., 2022. Kinetic theory and numerical simulation of biomineralization. *Chinese Journal of Geotechnical Engineering* 1-10 [In Chinese].

Zeng, H., Yin, L.Y., Tang, C.S., et al., 2020. Tensile behavior of bio-cemented, fiber-reinforced calcareous sand from coastal zone. *Engineering Geology* 294: 106390.

Gomez, M. G., B. C. Martinez, J. T. DeJong, C. E. Hunt, L. A. deVlaming, D. W. Major, and S. M. Dworatzek., 2014. Field-scale bio-cementation tests to improve sands. Proceedings of the ICE-Ground Improvement 168 (GI3):206-216.
